# Incorporating end-users’ voices into the development of an implant for HIV prevention: a discrete choice experiment in South Africa and Zimbabwe

**DOI:** 10.1186/s12905-023-02181-x

**Published:** 2023-02-10

**Authors:** Erica N. Browne, Kgahlisho Manenzhe, Wanzirai Makoni, Sikhanyisiwe Nkomo, Imelda Mahaka, Khatija Ahmed, Mary Kate Shapley-Quinn, Tozoe Marton, Ellen Luecke, Leah Johnson, Ariane van der Straten, Alexandra M. Minnis

**Affiliations:** 1 Women’s Global Health Imperative, RTI International, 2150 Shattuck Avenue, Suite 800, Berkeley, CA 94704 USA; 2 Setshaba Research Centre, Soshanguve, South Africa; 3 Pangaea Zimbabwe AIDS Trust (PZAT), Harare, Zimbabwe; 4 Faculty of Health Sciences, Department of Medical Microbiology, University of Pretoria, Pretoria, South Africa; 5 Biomedical Technologies Group, RTI International, Research Triangle Park, USA; 6 ASTRA Consulting, Kensington, USA; 7 Center for AIDS Prevention Studies, Department of Medicine, University of California San Francisco, San Francisco, USA

**Keywords:** Pre-exposure prophylaxis, Women, Discrete choice experiment, Implant, Sub-Saharan Africa, HIV

## Abstract

**Background:**

Input from end-users during preclinical phases can support market fit for new HIV prevention technologies. With several long-acting pre-exposure prophylaxis (PrEP) implants in development, we aimed to understand young women’s preferences for PrEP implants to inform optimal design.

**Methods:**

We developed a discrete choice experiment and surveyed 800 young women in Harare, Zimbabwe and Tshwane, South Africa between September–November 2020. Women aged 18–30 years who were nulliparous, postpartum, or exchanged sex for money, goods or shelter in prior year were eligible; quotas were set for each subgroup. The DCE asked participants to choose between two hypothetical implants for HIV prevention in a series of nine questions. Implants were described by: size, number of rods and insertion sites, duration (6-months, 1-year, 2-years), flexibility, and biodegradability. Random-parameters logit models estimated preference weights.

**Results:**

Median age was 24 years (interquartile range 21–27). By design, 36% had used contraceptive implants. Duration of protection was most important feature, with strong preference for a 2-year over 6-month implant. In Zimbabwe, the number of rods/insertion sites was second most important and half as important as duration. Nonetheless, to achieve an implant lasting 2-years, 74% were estimated to accept two rods, one in each arm. In South Africa, preference was for longer, flexible implants that required removal, although each of these attributes were one-third as important as duration. On average, biodegradability and size did not influence Zimbabwean women’s choices. Contraceptive implant experience and parity did not influence relative importance of attributes.

**Conclusions:**

While duration of protection was a prominent attribute shaping women’s choices for PrEP implants, other characteristics related to discreetness were relevant. Optimizing for longest dosing while also ensuring minimal detection of implant placement seemed most attractive to potential users.

## Background

Despite important advances in prevention, HIV remains a global public health priority, with an estimated 1.5 million new infections in 2020. In sub-Saharan Africa, women and girls accounted for 63% of new infections, with young women aged 15–24 twice as likely as young men to be living with HIV [1]. Long-acting pre-exposure prophylaxis (PrEP) shows promise as a new option in the HIV prevention toolbox. PrEP is highly efficacious at preventing HIV infection when taken as prescribed, but the currently available formulation as a daily tablet has been challenging for some, minimizing its effectiveness [2]. In particular, daily pill-taking has been a challenge for young women in African countries because of individual, relational, and structural factors as well as distribution interruptions [3–5]. Additional prevention tools with alternative delivery mechanisms offer hope for longer lasting protection with more convenience. Injectable cabotegravir (CAB-LA) was found to be an effective form of PrEP in cisgender women when given every two months and was recently approved by the US FDA [6, 7]. The monthly dapivirine vaginal ring was approved for use by the Medicines Control Authority of Zimbabwe in July 2021, with regulatory reviews in other African countries underway [8]. While both of these upcoming products are longer-acting, they may still require frequent clinic visits. Another delivery platform, the implant, provides another option for increased convenience through longer duration of protection. Currently, there are several PrEP implant technologies in development [9–14].

Embedding end-users’ perspectives throughout the research and development process is critical to ensuring new prevention products meet the needs and interests of the intended users [15]. Typically, acceptability research is performed within the context of clinical trials. However, opinions from trial participants may not be representative of the target end-user population, given their willingness to take part in a trial of a novel biomedical technology [16]. In addition, assessing acceptability in late-stage trials may leave little opportunity to make product design alterations based on end-users’ perspectives. To evaluate the potential market fit for a new prevention technology, input from target users should be collected in pre-clinical phases that afford opportunities to inform product development.

Stated preference surveys, particularly discrete choice experiments (DCEs), have become frequently used in health care to evaluate the relative importance of specific aspects of products or services not yet available [17–21]. Previous qualitative research has explored women’s preferred characteristics for an HIV prevention implant or multipurpose prevention technology (MPT) implant that prevents both HIV and pregnancy [22–24]. These data provide useful insights into end-users’ initial perceptions of the product, their preferences, and the contexts which shape their preferences. However, it may not be possible to incorporate all preferred characteristics noted by end-users into a single product, and developers may have to forgo one favored feature of the product to improve another. Other research techniques are therefore needed to help developers decide which features to prioritize to maximize potential for the final product to be acceptable and desirable to end-users. DCEs assume that healthcare products can be described by distinct features or attributes, and that the value of the product depends upon the options or levels of these attributes. The participant is asked to choose between products defined by the same attributes although with different levels, to understand the relative importance of the product’s attributes. By observing the decisions end-users make between alternative implant designs, we can understand which attributes or features of an implant are relatively more important than others and the preferred levels.

In this study, we aimed to gather feedback on implant preferences from a large sample of young women in South Africa and Zimbabwe using a DCE. We developed and surveyed a DCE with young women, a key target user group, to understand which attributes of the implant should be prioritized and what tradeoffs among attributes women would be willing to make. Providing input from potential end-users while implants are still early in the development process holds promise to optimize the product design and support successful clinical trials and ultimate roll-out of effective technology.

## Methods

### Sample and study procedures

Women who were between the ages of 18 and 30 years and met at least one of the following criteria were eligible to participate: [1] had never given birth, (2) had given birth in the past 18 months, (3) had exchanged sex for money, goods or shelter in the past 12 months. Enrollment quotas were set for each of these three enrollment criteria subgroups (n ≥ 100 per enrollment location). In addition, an enrollment target was set based on experience ever using a contraceptive implant (n = 100 per location). These criteria were created to ensure a diverse range of perspectives from women considered to be candidate users of an HIV prevention implant. The complexity of the DCE design (number of attributes and question format) as well as the desire to be able to test for differences between subgroups informed the sample size of 800, with 400 participants per site to ensure balanced representation across enrollment locations [25].

The study design and data collection materials were developed and implemented as a collaboration between Pangea Zimbabwe AIDS Trust (PZAT), Setshaba Research Centre (SRC), and RTI International. Recruitment occurred between September and November 2020 in Harare, Zimbabwe and Tshwane, South Africa. Various strategies were implemented to recruit the diverse range of participants, including community-, clinic- and social media-based approaches (e.g., Facebook and WhatsApp announcements). In Harare, PZAT’s core strategies included partnership with local organizations to conduct recruitment through their clinic and mobile site networks in several high-density suburbs. In Tshwane, women were recruited from community engagement at malls, social grant collection centres, family planning clinics, alongside partnership with Ministry of Health clinics and other local organizations. Staff described study participation as an opportunity to contribute to co-designing a future HIV prevention option for women, in that scientists wanted to incorporate women’s preferences into new HIV prevention technology. Women were invited to complete the approximately 1-h survey at the study clinic. Interviewers administered the survey on tablet computers following COVID-19 public health safety protocols. Participants were reimbursed approximately $3 US in local currency. All study procedures were reviewed and approved by the Medical Research Council of Zimbabwe and Pharma-Ethics Committee in South Africa; all participants provided written informed consent prior to enrollment.

### Discrete choice experiment and survey instrument development

**Fig. 1:**
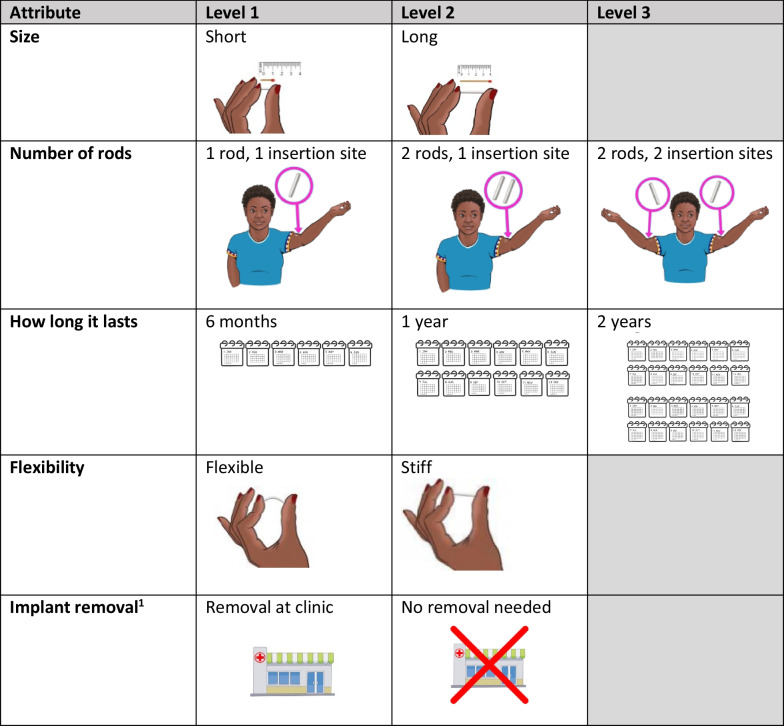
List of implant attributes with corresponding levels included in the discrete choice experiment. ^1^Implant removal attribute assessed preference for whether the implant requires removal at the clinic by a health care provider versus dissolves on its own and does not need to be removed once all medicine has been released (made from biodegradable polymer). The attribute description stated that all implants can be removed by a health care provider after they are inserted, either for safety reasons or if the woman using the implant decides she no longer wants it

The attributes and levels used to describe implants in the DCE were selected based on prior exploratory qualitative research with end users in these geographic locations and tradeoff decisions identified by the product development team as priorities to guiding the implant design. We focused on modifiable characteristics and those for which tradeoffs may be needed (e.g., flexibility, rod length, duration, and number of rods). The five attributes with their corresponding levels are outlined in Fig. 1. Attribute descriptions and images were pre-tested in cognitive interviews to ensure clarity and cultural relevance of attribute descriptions and images. Before starting the survey, participants watched a short video where study staff briefly explained the concept of an implant for HIV prevention and introduced each attribute: rod length, number of rods and insertion sites on the body, duration, flexibility, and biodegradability (described as need for removal versus dissolves on its own and does not need to be removed once all medicine has been released). The survey then introduced each attribute in more detail; illustrations were included for each level to assist participants’ comprehension. In addition, participants handled placebo prototypes (in plastic bags) exemplifying the different options for implant designs to help limit potential bias from the hypothetical nature of the exercise.

**Fig. 2:**
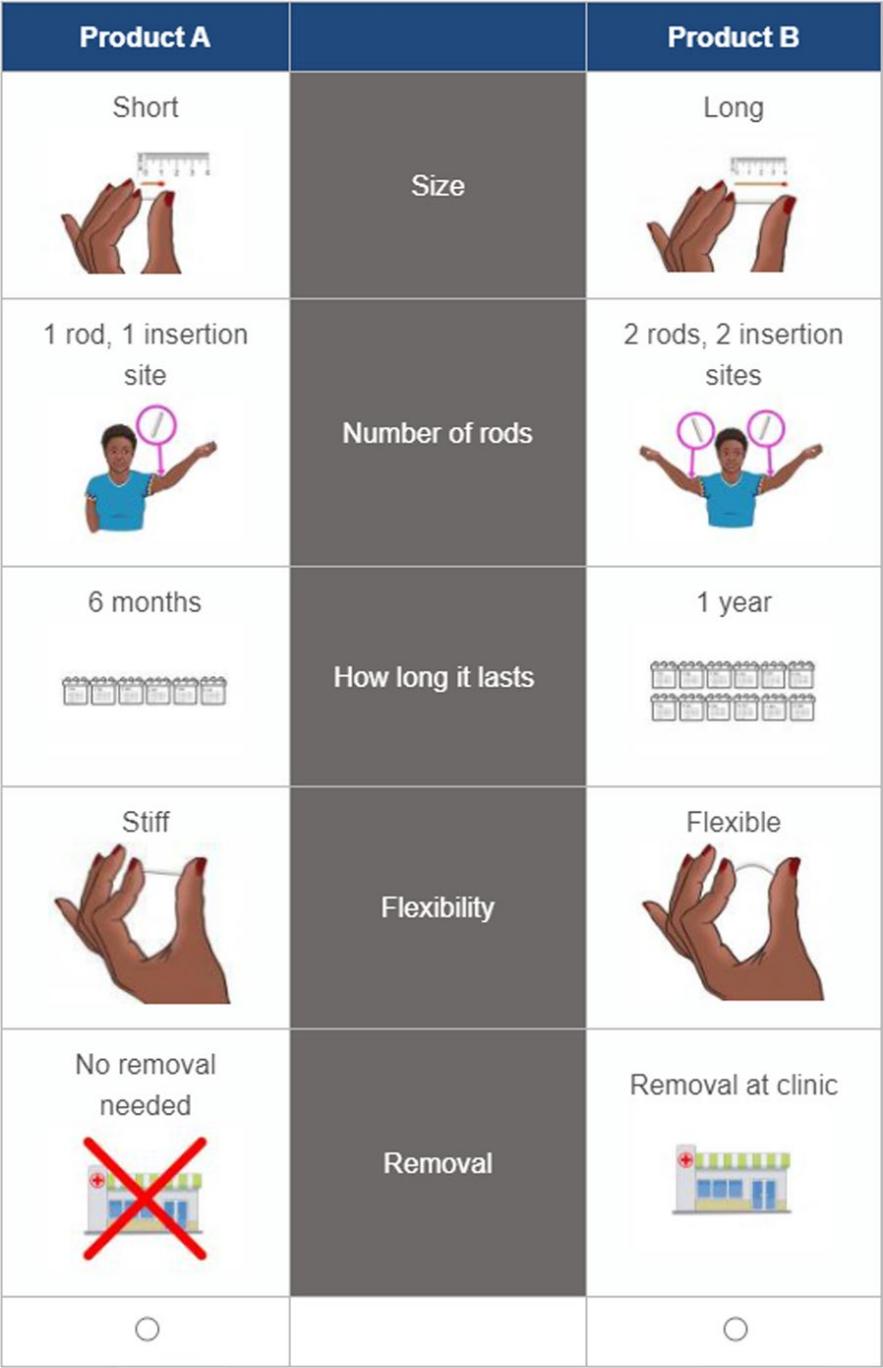
Example choice set in discrete choice experiment survey. Participants were asked: “Which product would you use for HIV prevention?”

The DCE portion of the survey had participants choose which implant they would use for HIV prevention between pairs of hypothetical implants (see choice task example in Fig. 2). The combinations of attribute levels used to describe each implant were created using an experimental design following good research practices [19]. A D-efficient algorithm was used in SAS 9.4 (SAS Institute, Cary NC) to construct a fractional factorial design, which produced 72 choice task questions that were then split into eight blocks of nine questions each.

Participants were randomly assigned to complete one of the eight blocks, ensuring equal number of participants completed each block across both clinics. After each choice set question, participants were asked “If the product you chose was available, do you think you would actually use it?” to gauge interest given choice response was required (opt-out). Following the choice tasks, participants were asked additional questions about preferences for HIV prevention implants and completed a behavioral and demographic questionnaire. The survey was programmed using Qualtrics software (Qualtrics, Provo, UT) and offered in English, Shona, and Tswana.

### Statistical analyses

Before beginning analysis, we assessed whether preference data from the two countries were on the same scale and therefore could be pooled together [26]. The Swait and Louviere test indicated that there were differences in preferences by country (Χ^2^ = 44.47, *p* < 0.01) which confounded the ability to test for scale differences; therefore, choice data from each enrollment location were modelled separately.

Choice task data from the DCE were analyzed using a random parameters logit (RPL) model. RPL models are typically used to analyze preference data because they incorporate heterogeneity by estimating a normal distribution for each attribute level parameter [27]. Attribute levels were included in the model as effects-coded variables, whereby the mean effect for each attribute was normalized at zero. Normalized preference weights (PW) derived from RPL models represent the mean importance of an attribute level relative to other levels presented. A larger more positive weight indicates relatively more preference than average and a smaller negative weight indicates less preference. The difference between the largest and smallest preference weight within each attribute indicates the relative importance (RI) of the attribute to the decision-making process. For ease of interpretation, RI scores were rescaled on a range of 0–10 with a score of 10 applied to the most important attribute and other RI scores scaled relative to most important [10]. PW were also used to estimate preference shares, or the probability that the average participant would select one implant design over a set alternative. We hypothesized preferences may differ by demographics, parity, history of engaging in transactional sex, and implant experience; therefore, we tested for differences in preferences using fixed interaction terms between all attribute levels and a binary indicator for the subgroup in the main RPL model. We tested for differences in likelihood of opting-out by implant experience using a mixed-effect logistic regression model, adjusting for enrollment location and including a random intercept for participant to account for repeated measures. Descriptive statistics (i.e. means, frequencies) were used to summarize demographic and behavioral characteristics of participants and additional preference questions. All analyses were completed using Stata 16.0 (StataCorp, College Station, Texas).

The funder did not have any role in the design, interpretation, writing, or publishing of this research.

## Results

The median age of participants was 24 years (interquartile range 21–27). Participants’ sociodemographic and behavioral characteristics varied by enrollment location (Table 1). More women in Tshwane, South Africa had completed high school (74% versus 36% in Harare, Zimbabwe) and were currently in school (35% versus 11%), and fewer earned an income (10% versus 40% in Zimbabwe). Nearly two-thirds of participants described their current household financial situation as difficult (50%) or very difficult (12%) and that access to money for necessary items had decreased because of COVID-19 (62%). Most (79%) currently had a primary partner; more women in Zimbabwe were married or living with their partner (44% versus 10% in South Africa). By design, approximately a third of participants enrolled at each site had experience using a contraceptive implant; 22% were currently using an implant (17% in South Africa and 27% in Zimbabwe). Most women with implant experience (63%) had used an implant for at least one year. Three-quarters noted being “satisfied” (36%) or “very satisfied” (38%) with using a contraceptive implant. Those who were no longer using a contraceptive implant (n = 112) noted side effects (59%) and changes to menstruation (25%) as main reasons for discontinuing.

**Table 1:** Characteristics of participants, overall and by enrollment location (Sept – Nov 2020)

	Tshwane, South Africa		Harare, Zimbabwe		Total	
	N	(%)	N	(%)	N	(%)
Total	400	(100)	400	(100)	800	(100)
Age—mean, median (interquartile range)	23.3, 23	(20–26)	24.4, 24	(22–27)	23.8, 24	(21–27)
Completed Matric/High School	296	(74)	140	(36)	436	(54)
Currently in school	138	(35)	42	(11)	180	(23)
Earn an income	42	(10)	160	(40)	202	(26)
Current household financial situation "difficult/very difficult"	252	(63)	256	(64)	498	(62)
Food insecure^b^	213	(53)	184	(46)	397	(50)
Currently have a primary partner	337	(84)	291	(73)	628	(79)
Married or living with a partner	19	(5)	174	(44)	193	(24)
Partner provides financial or material support	273	(68)	270	(68)	543	(68)
*Primary partner had other partners past 6 months*						
Yes, I know or think so	75	(19)	76	(19)	151	(19)
Don’t know	171	(43)	179	(45)	350	(44)
Ever exchanged sex	146	(37)	108	(27)	254	(32)
Exchanged sex in past year^a^	101	(25)	100	(25)	201	(25)
*Number sex partners in past 6 months*						
0	26	(7)	68	(17)	94	(12)
1	256	(64)	228	(57)	484	(61)
2 or more	117	(30)	104	(26)	221	(22)
Condom used at last sex	191	(48)	133	(35)	324	(42)
*Contraceptive method(s) ever used*						
Male condom	303	(76)	200	(50)	503	(63)
Injectables	245	(61)	69	(17)	314	(39)
Pills	122	(31)	209	(52)	331	(41)
Implant^a^	131	(33)	158	(40)	289	(36)
IUD	19	(5)	18	(5)	37	(5)
Currently using contraceptive method	282	(71)	291	(73)	573	(72)
Nulliparous^a^	322	(40)	238	(30)	560	(35)
*Current method(s) for HIV prevention*						
Male condoms	259	(65)	132	(33)	391	(49)
Oral pre-exposure prophylaxis (PrEP)	11	(3)	23	(6)	34	(4)
Partner testing/know partner is HIV negative	97	(24)	59	(15)	156	(20)
Last HIV test > 6 months ago	106	(27)	163	(41)	269	(34)
Agree "my sexual behavior gives me a chance of getting HIV"	124	(31)	155	(39)	279	(35)
Agree "my partner will accuse me of being unfaithful if I want to use an HIV prevention method"	136	(35)	130	(32)	266	(33)

^a^Enrollment quota

^b^Worried would not have enough food 3 or more time in past 4 weeks

Nearly half of participants (49%) reported currently using male condoms for HIV prevention; 4% (n = 34) were currently using oral pre-exposure prophylaxis.

**Fig. 3:**
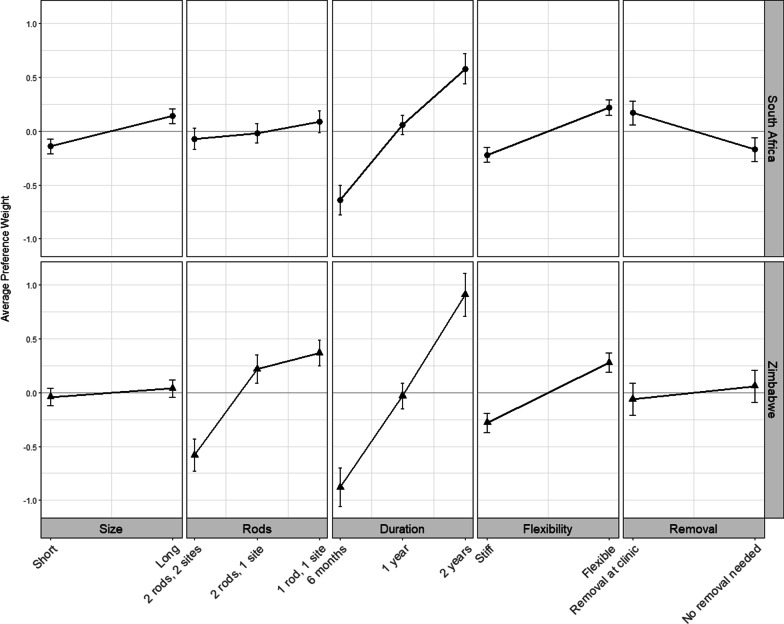
Normalized average preference weights estimated using random parameter logit models, per enrollment location

Because preferences were found to differ by country (*p* < 0.01), separate models were estimated, and the average preference weights for each RPL model are depicted graphically in Fig. 3. In both locations, duration was the most important attribute in choosing between implant designs, with 2-years strongly preferred over a 1-year and 6-month (least preferred) implant. Among young women in South Africa, there was no clear second most important attribute. They had preferences among levels of other attributes, but each attribute overall was roughly one-third as important as duration. On average they preferred that an implant be flexible (PW: 0.22, 95% CI 0.14, 0.29) instead of stiff, long (PW: 0.14, 95% CI 0.07, 0.21) versus short, and that an implant be removed (PW: 0.17, 95% CI 0.07, 0.28) over biodegrade. The number of rods and insertions was the least important attribute to South African women’s decision making; preference weights for each of these combined levels were not significantly different from zero.

In contrast, the number of rods and insertion sites was the second most important attribute for Zimbabwean women and half as important as duration (Fig. 3). On average, Zimbabwean women preferred one insertion site, with little difference in preference for one rod (PW: 0.37, 95% CI 0.25, 0.49) versus two rods (PW: 0.22, 95% CI 0.09, 0.35), compared to two rods in two sites, which was less preferred (PW: − 0.58, 95% CI − 0.74, − 0.43). On average, they also preferred a flexible implant (PW: 0.28, 95% CI 0.18, 0.37) but did not have discernable preferences surrounding length (PW for long: 0.04, 95% CI − 0.03, 0.12) or whether the implant needed to be removed (PW: − 0.06, 95% CI − 0.21, 0.09) versus biodegrade.

Among the 7200 choices made by all 800 participants, 88% of chosen designs were implants that participants indicated they would actually use if available. In South Africa, nearly half of participants (48%) opted out at least once (i.e., indicated they would not actually use their chosen design); 15 (4%) opted out of ≥ 7 of their 9 choices. Fewer participants in Zimbabwe opted out of their chosen designs; 27% opted out at least once and only 2 participants (< 1%) opted out of ≥ 7 choices. Those with prior implant experience were less likely to opt-out (adjusted odds ratio 0.50, 95% CI 0.33, 0.77; *p* = 0.002). There was little evidence that choices were made based on preference for a single attribute; 10% of participants in both sites consistently chose the product with the longer duration.

Following the choice tasks, participants were asked directly about the importance of each attribute and to rank attributes from most to least important [data not shown]. About two-thirds rated each attribute as ‘very important’ when selecting an implant; 90% rated duration ‘very important.’ Overall, most participants ranked either duration (38%), implant length (23%), or number of insertions (16%) as most important. More South African women ranked length as most important (30% versus 15%, *p* < 0.001) and more Zimbabwean women ranked insertion sites as most important (23% versus 10%, *p* < 0.001). Biodegradability was frequently ranked least important (43%), although the majority still noted that it was a ‘somewhat’ (19%) or ‘very’ (68%) important factor when selecting an implant for HIV prevention.

### Preference shares

**Fig. 4:**
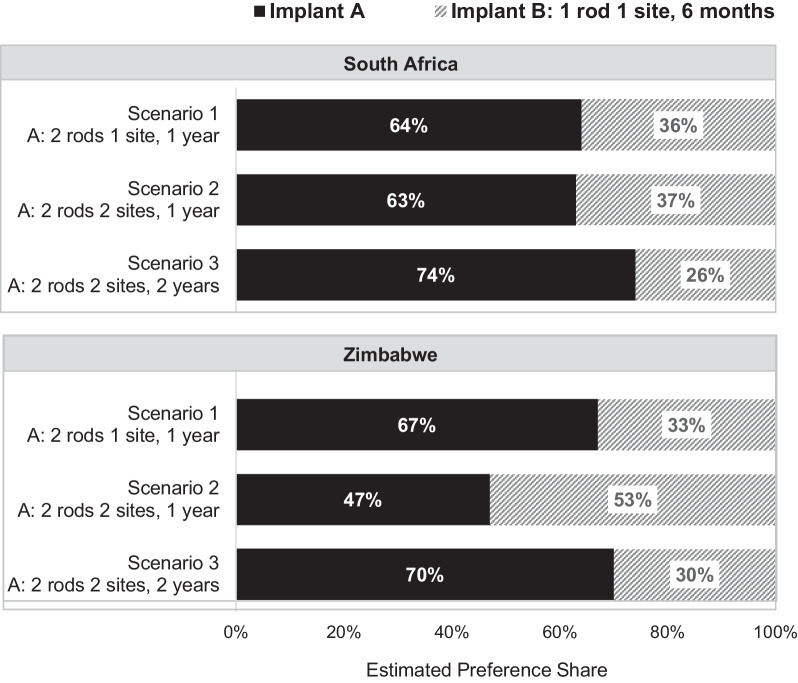
Preference share analysis for HIV prevention implant. Results from the random parameter logit models were used to predict the probability that the average participant in each geographic location would choose between two implants in three scenarios where Implant A has varying number of insertion sites and duration of protection and Implant B is 1 rod that lasts for 6 months (all other attributes held constant)

Preference weight estimates were used to calculate the probability of the average participant’s choice between two alternative implant designs. Figure 4 outlines by geographic location the share of the sample estimated to choose between two implants in three scenarios, Product A is a two-rod implant with varying number of insertion sites and years of duration and Product B is a 1 rod implant that lasts for 6 months (all other attributes held constant). In South Africa, an estimated larger share of participants would, on average, select the product that has a longer duration (Product A), regardless of the number of insertion sites. In Zimbabwe, fewer women were estimated to choose the longer acting implant if it required two rods in two insertion sites (Scenario 2) because on average, they were not willing to have an additional incision to gain 6 months of additional protection. However, if an implant requires two rods and two incisions but offers substantially longer duration of protection (2 years instead of 6 months, Scenario 3), then a larger share of women was estimated to be willing to accept two incisions to gain 1.5 additional years of protection.

### Preference heterogeneity

Beyond geographic location, we assessed whether there was heterogeneity in preferences explained by younger age, education, prior implant experience, parity, history of transactional sex. For all subgroups, the relative importance of attributes remained comparatively the same, with duration most important. In both locations, women who had experience using a contraceptive implant had on average more preference for a long-4 mm implant compared to short-1 mm implant (Zimbabwe PW difference: 0.19, 95% CI 0.04, 0.35, *p* = 0.02; South Africa PW difference: 0.14, 95% CI − 0.01, 0.29, *p* = 0.06). In addition, Zimbabwean women who were implant-experienced were more willing to accept two rods in a single location compared to implant-naïve participants (PW difference: 0.31, 95% CI 0.05, 0.57; *p* = 0.02). Similarly, parous women in both locations had stronger preference for a longer versus shorter implant; they also had less preference surrounding flexibility and number of rods. Of note, implant experience was highly correlated with parity. Only 4% of Zimbabwean women (n = 7) and 18% of South African women (n = 23) who were implant-experienced were nulliparous. There were no significant differences in preferences among those who had engaged in transactional sex in the past year or by age. Zimbabwean women who had completed high school had stronger preference for a flexible implant (PW difference: 0.35, 95% CI 0.16, 0.53; *p* < 0.001) such that flexibility was nearly equally as important to decision making as rods/insertion sites.

## Discussion

In a DCE with young women in Zimbabwe and South Africa who would be candidate users of a long-acting HIV PrEP implant, the majority of implant designs were found to be acceptable, with most women indicating that they would use their chosen implants. Women prioritized duration of protection when deciding between implant designs, with a strong preference for a 2-year over 6-month implant. Zimbabwean women’s choices were also influenced by the number of insertion sites, where if the implant technology required two rods, the preference was for both rods to be inserted in the same location instead of one in each arm. While less important to decisions, on average, women also preferred a flexible over stiff implant. They also preferred or were willing to accept a longer (4 mm) instead of shorter (1 mm) implant. Preferences for biodegradability were mixed and on average, considered relatively less important compared to other implant features. While we found there was some heterogeneity in preference based on prior implant experience and parity, it was not substantial enough to modify the relative importance of attributes.

Similar to other PrEP studies, implant choices were primarily shaped by preference for less frequent dosing [22, 28–32]. Although women noted that several other implant features were also important and, when asked directly, two-thirds indicated a feature other than duration was most important, women were ultimately not willing to forgo 6-months of protection in order to have other desired characteristics. In a qualitative study, preference for longer-acting implants was attributed to desire for reduced clinic visits and less burden of remembering to use a product [22]. Similarly, health care providers have expressed that long duration is the most important attribute, as longer-lasting implants would decrease burden on the health care system, reduce costs, and be more convenient for patients. They referenced women’s adherence challenges with contraceptive injections as evidence for needing implants to last as long as possible [33].

The number of insertion sites and the physical flexibility of the implant were other important attributes to selection of an implant, potentially for their connection to discreetness. For Zimbabwean women, the number of insertion locations was half as important as duration. Aversion to having two incisions, one in each arm, may be related to fears of additional pain and scarring from placement [23, 34]. However, most were willing to accept two incisions if it meant they would have two years of protection. South African women on average found that the difference between two rods inserted in one or two sites was not as important as other implant features, and they were willing to trade the number of incisions for a more flexible implant. The desire for discreetness has been described as the reasoning behind flexibility preference [22, 33, 35]. Increased flexibility implies decreased palpability which in turn makes the implant less visible. In another study, youth in South Africa noted invisibility, or the ability to use a method without anyone knowing, as the main reason why long-acting prevention methods like injectables and implants were more attractive than oral pills or vaginal rings [35]. While health care providers require some level of palpability for removal, product developers should consider how palpability influences visibility under the skin. Furthermore, attempt to optimize design for minimal detection of implant placement will be important and could be achieved via more discreet body sites than the arm, alternative product designs and geometries and use of low rigidity materials.

Biodegradability was considered the least important attribute among those evaluated, with mixed preferences. On average South African women preferred that the implant be removed and Zimbabwean women on average had no preference around biodegradability. The concept of biodegradability, where medicine is still being released as the device is breaking down, might be challenging for end-users to understand. In previous contraception research, most women expressed concern about where the contents of an implant go as it dissolves and the potential health effects related to biodegradation [35, 36]. This concern may have influenced preference for removal even though that would require another surgical procedure. Rare, documented cases and existing myths surrounding contraceptive implants “disappearing” in the body might impact opinions of a dissolving implant for HIV prevention. Contraceptive implants were more recently introduced in South Africa (in 2014) and uptake has declined following challenges with introduction that impacted perception of implants [37]. Limited awareness and knowledge of implants was previously associated with low uptake of the method [38, 39]. Therefore, innovative technologies like a biodegradable implant, which provides the opportunity for increased convenience for the user, likely will require sensitization and educational efforts to dispel rumors and misconceptions and ultimately, be found acceptable [36, 40].

There are some limitations to this study. Our findings are likely not representative of all women who would benefit from using an implant for HIV prevention, given women self-selected to participate, recruitment only occurred in urban/suburban areas, and that we had targeted quotas based on parity, history of exchanging sex, and implant experience. Therefore, our study is limited in understanding overall demand for HIV prevention implants. However, our intention was to understand preferences from these specific potential user groups, given their heightened susceptibility to HIV [41], and our sample likely reflects those most interested in using implants. Discrete choice experiments are hypothetical in nature, and participants’ stated preferences may not align with actual choices. But when products are not yet available, this methodology provides a means for measuring preferences that more closely resembles real-world decision making and offers insights regarding users’ preferences to inform product development early in the pipeline [42]. Given DCEs estimate data about relative attribute preferences, other implant attributes not included in our design, such as cost, could be informative in shaping preferences. Therefore, introduction of attributes pertinent to other aspects of implant design and delivery or additional attribute levels could shift ordering of attribute preferences. Finally, study implementation during the COVID-19 pandemic may have influenced who was able to participate and general interest in pre-exposure prophylaxis. 

## Conclusion

Overall, young women in South Africa and Zimbabwe expressed interest in using at least one of the implant designs presented for HIV prevention. How long the implant provides protection was most important in shaping interest. However, other characteristics related to discreetness were still considered when deciding between options. Attribute priorities were consistent between nulliparous and parous women and between women with and without contraceptive implant experience. To develop an implant with high potential acceptability, developers should consider optimizing for longest dosing while also ensuring other features maintain minimal detection of implant placement and discreetness.

## Data Availability

The datasets used and/or analyzed during the current study are available from the corresponding author on reasonable request.
